# Simultaneous Spectrophotometric Estimation of Norfloxacin and Ornidazole in Tablet Dosage Form

**DOI:** 10.4103/0250-474X.56026

**Published:** 2009

**Authors:** S. B. Wankhede, A. Prakash, B. Kumari, S. S. Chitlange

**Affiliations:** Department of Pharmaceutical Chemistry, Pad. Dr. D. Y. Patil Institute of Pharmaceutical Sciences and Research, Sant Tukaram Nagar, Pimpri, Pune-401 018, India

**Keywords:** Norfloxacin, ornidazole, simultaneous equation, Q-analysis, derivative spectroscopy

## Abstract

Three simple, accurate and economical methods have been developed for the estimation of norfloxacin and ornidazole in tablet dosage form. First method is based on the simultaneous equations, wavelengths selected for analysis were 273.0 nm (λ_max_ of norfloxacin) and 318.5 nm (λ_max_ of ornidazole), respectively, in 0.1N NaOH. Second method is Q-analysis method, based on absorbance ratio at two selected wavelengths 297.0 nm (iso-absorptive point) and 318.5 nm (λ_max_ of ornidazole). Third method is first order derivative spectroscopy using 297.5 nm (zero cross for norfloxacin) and 264.0 nm (zero cross for ornidazole). The linearity was obtained in the concentration range of 4-20 μg/ml and 5-25 μg/ml for norfloxacin and ornidazole, respectively. The results of the analysis have been validated statistically and by recovery studies.

Norfloxacin (NF), chemically 1-ethyl-6-fluoro-1,4-dihydro-4-oxo-7-(1-piperazinyl)-3-quinoline carboxylic acid[1], is a synthetic broad spectrum fluoroquinolone antibacterial agent used in the treatment of urinary and genital tract infection[2,3]. Ornidazole (OZ), chemically 1-chloro-3-(2-methyl-5-nitro-imidazol-1-yl) propan-2-ol, is an antimicrobial agent used in treatment of susceptible protozoal infections and anaerobic bacterial infection[4,5]. NF is official in USP[1], BP[6] and IP[7] whereas OZ is not official in any pharmacopoeia. Both the drugs are marketed as combined dose tablet formulation in the ratio of NF:OZ 400:500 mg. Literature survey revealed that a number of methods have been reported for estimation of NF[8–11] and OZ[12–17] individually or in combination with other drugs. However, there is no analytical method reported for the simultaneous estimation of norfloxacin and ornidazole in a combined dosage formulation. Present work describes three simple, accurate, reproducible, rapid and economical methods for simultaneous estimation of NF and OZ in tablet formulation.

A double-beam Shimadzu UV/Vis spectrophotometer, 1700 Pharmaspec, with spectral bandwidth of 2 nm, wavelength accuracy of ±0.5 nm and a pair of 1-cm matched quartz cells, was used to measure absorbance of the resulting solution. Standard gift sample of norfloxacin was provided by Emcure Pharmaceuticals Ltd., Pune and ornidazole by Aristo Pharmaceuticals Pvt. Ltd., Mumbai. Combined dose NF and OZ tablets (Norrit-Ord, 400 mg norfloxacin and 500 mg ornidazole; Ind-Swift Ltd., Chandigarh), were purchased from the local pharmacy. Sodium hydroxide, 0.1N, was prepared from analytical reagent grade sodium hydroxide in double distilled water and used as a solvent. Standard stock solutions of NF (100 μg/ml) and OZ (100 μg/ml) were prepared and used for the analysis.

For the selection of analytical wavelength for the simultaneous equation method (Method-A), solutions of NF and OZ (20 μg/ml, each), were prepared separately by appropriate dilution of standard stock solution and scanned in the spectrum mode from 200 nm to 400 nm. From the overlain spectra of both drugs (fig. 1), wavelengths 273.0 nm (λ_max_ of NF) and 318.5 nm (λ_max_ of OZ) were selected for the simultaneous equations. The calibration curves for NF and OZ were prepared in the concentration range of 4-20 μg/ml and 5-25 μg/ml at both the wavelengths respectively. The absorptivity values were determined for both the drugs at both the wavelengths and following Eqns were used, A_1_ = 110.9C_NF_+15.3C_OZ_ (1) and A_2_ = 43.7C_NF_+28.4C_OZ_ (2), where A_1_ and A_2_ are absorbances of the sample at 273 nm and 318.5 nm, respectively, 110.9 and 43.7 are absorptivities of NF at 273.0 and 318.5 nm, respectively, 15.3 and 28.7 are the absorptivities of OZ at 273.0 nm and 318.5 nm, respectively. C_NF_ is the concentration of NF and C_OZ_ is the concentration of the OZ. The mixture concentration was determined by using the Eqns. 1 and 2.

**Fig. 1:**
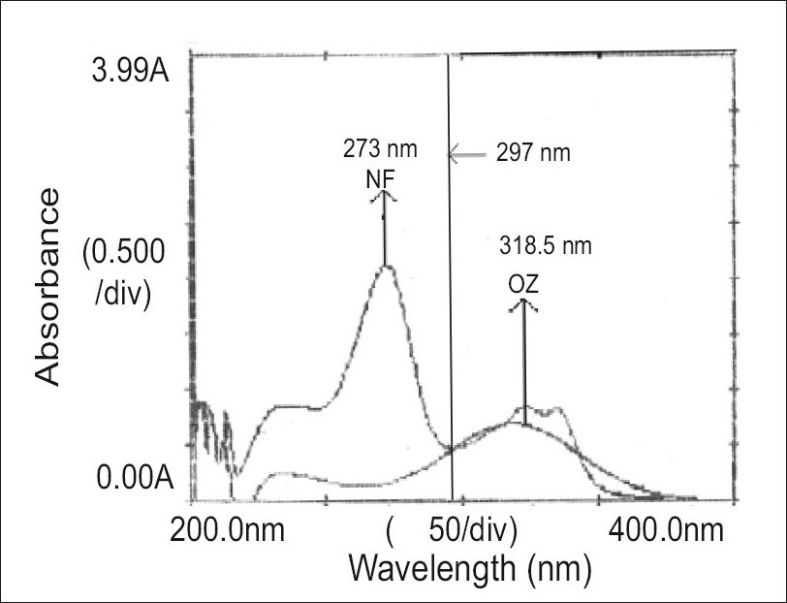
Overlain spectra of norfloxacin and ornidazole. Overlain spectra of norfloxacin (NF, 273 nm) and ornidazole (OZ, 318.5 nm)

In the absorption ratio method (Method-B), from the overlain spectra of both drugs (fig. 1), wavelengths 297.0 nm (iso-absorptive point) and 318.5 nm (λ_max_ of OZ) were selected for the analysis. The calibration curves for NF and OZ were plotted in the concentration range of 4-20 μg/ml and 5-25 μg/ml at both the wavelengths respectively. The absorptivity values were determined for both the drugs at both the wavelengths. From the following set of Eqns the concentration of each component in the sample can be calculated, Cx = Qm–Qy/Qx–Qy×A_1_/a (1) and Cy = Qm–Qx/Qy–Qx×A_1_/a (2), where Cx is the concentration of NF, Cy is the concentration of OZ, A_1_ is the absobance of sample at iso-absorptive wavelength 297.0 nm, a is the mean absorptivity of NF and OZ at iso-absorptive wavelength 297.0 nm, Qm is the ratio of absorbance of sample solution at 318.5 nm and at 297.0 nm, Qx is the ratio of absorptivities of NF at 318.5 nm and at 297.0 nm and Qy is the ratio of absorptivities of OZ at 318.5 nm and at 297.0 nm.

In first order derivative spectroscopy (Method-C) solutions of NF and OZ (20 μg/ml, each), were prepared separately by appropriate dilution of standard stock solution and scanned in the spectrum mode from 200 nm to 400 nm. The absorption spectra thus obtained were derivatized from first to fourth order. First order derivative spectrum was selected for analysis of both drugs. The zero crossing wavelengths 297.5 nm (zero cross for NF) and 264.0 nm (zero cross for OZ) were selected for the analysis. The calibration curves for NF and OZ were plotted in the concentration range of 4-20 μg/ml and 5-25 μg/ml at both the wavelengths, respectively. The concentration of the individual drug present in the mixture was determined against the calibration curve in quantitation mode.

For the estimation of drugs in the commercial formulations, twenty tablets were weighed and average weight was calculated. The tablets were crushed to obtain fine powder. Tablet powder equivalent to 80 mg of NF was transferred to 100.0 ml volumetric flask containing 40 ml of 0.1N NaOH and ultrasonicated for 10 min and diluted to the mark with 0.1N NaOH. The solution was then filtered through a Whatmann filter paper No. 41. From the filtrate 5.0 ml was transferred to a 50.0 ml volumetric flask and diluted to the mark with 0.1N NaOH to obtain 8 μg/ml of NF and 10 μg/ml of OZ. The concentration of both NF and OZ was determined by measuring the absorbance of the sample at 273.0 nm and 318.5 nm (Method-A) and at 297.0 nm and 318.5 nm (method B) in the spectrum mode and values were substituted in the respective formulae to obtain concentrations. For Method-C concentration of both NF and OZ was determined by measuring the absorbance of the sample at 297.5 nm and 264.0 nm in first order spectrum mode. The results of the tablet analysis were calculated against the calibration curve in quantitation mode.

Recovery studies were carried out by standard addition method at three different levels 80%, 100% and 120%. The % recovery of NF and OZ in the sample mixture was determined. The results of tablet analysis and recovery studies obtained by proposed method were validated by statistical evaluation and are recorded in Tables 1 and 2.

**TABLE 1:** ANALYSIS OF TABLET FORMULATION

Method	Component	Label Claim (mg/tab)	Amount Found	Estimated Label Claim*	SD	CV
A	NF	400	398.89	99.73	0.5932	0.5948
	OZ	500	496.60	99.32	0.8720	0.8780
B	NF	400	396.51	99.13	0.6855	0.6915
	OZ	500	498.89	99.78	0.7358	0.7374
C	NF	400	400.10	100.03	0.7842	0.7840
	OZ	500	498.62	99.68	0.9286	0.9316

* Average of six determinations, SD is standard deviation and CV is coefficient of variation.

**TABLE 2:** RESULTS OF RECOVERY STUDIES

Level of Recovery (%)	Amt. of Pure		Method-A		Method-B		Method-C	
			
	Drug Added (mg)		% Recovery		% Recovery		% Recovery	
				
	NF	OZ	NF	OZ	NF	OZ	NF	OZ
80	64	80	99.41	99.98	98.32	100.80	99.49	99.49
100	80	100	99.65	99.94	98.49	100.91	99.22	99.55
120	96	120	99.80	99.65	98.57	100.76	100.31	99.57
Mean % Recovery			99.62	99.86	98.46	100.82	99.81	99.53
SD			0.2147	0.2910	0.1578	0.2231	0.6711	0.7216
CV			0.2155	0.2914	0.1603	0.2213	0.6724	0.7250
SE			0.0877	0.1188	0.0644	0.0911	0.2740	0.2946

SD is standard deviation, CV is coefficient of variation and SE is standard error.

The methods discussed in the present work provide a convenient and accurate way for simultaneous analysis of NF and OZ. Percent label claim for NF and OZ in tablet, by all the methods, was found in the range of 98.15% to 101.03%. Standard deviation and coefficient of variance for six determinations of tablet sample, by both the methods, was found to be less than ±2.0 indicating the precision of both the methods. Accuracy of proposed methods was ascertained by recovery studies and the results are expressed as % recovery. Percent recovery for NF and OZ, by all three methods, was found in the range of 98.27% to 101.07%, values of standard deviation and coefficient of variation were in the range of ±0.1578 to ±0.7216 and 0.1603 to 0.7250, respectively indicating the accuracy of proposed methods. Based on the results obtained, it is found that the proposed methods are accurate, precise, reproducible and economical and can be employed for routine quality control of norfloxacin and ornidazole in combined dose tablet formulation.

## References

[1] (2003). The United State Pharmacopoeia.

[2] Petri WA, Hardman JG, Limbird LE, Gilman AG (2001). Antimicrobial agents: Sulfonamides, trimethoprimsulfamethoxazole, quinolones and agents for urinary tract infections. Goodman and Gillman's the pharmacological basis of therapeutics.

[3] Tripathi KD (2004). Essentials of medical pharmacolgy.

[4] Sweetman SC (2002). Martindale, The complete drug reference.

[5] Budavari S (1996). The Merck Index.

[6] (2007).

[7] (1996). Indian Pharmacopoeia.

[8] More HN, Mahadik KR, Kadam SS (1994). Simultaneous estimation of Norfloxacin and Tinidazole using UV visible spectrophotometer. Indian Drugs.

[9] Shrinivas Reddy GK, Jain DK, Trivedi P (1999). Derivative spectrophotometric and graphical absorbance ratio method for simultaneous estimation of norfloxacin and tinidazole in two component tablet formulation. Indian J Pharm Sci.

[10] Mahareshwari RK, Chaturvedi SC, Jain NK (2006). Novel spectrophotometric estimation of some poorly water soluble drugs using hydrotropic solubilizing agents. Indian J Pharm Sci.

[11] Argekar AP, Kapadia SP, Raj SV (1996). Simultaneous determination of norfoxacin and tinidazole in tablets by reverse phase-high performance liquid chromatography. Anal Lett.

[12] Kasture VS, Bhagat AP, Puro NC (2004). Spectrophotometric method for simultaneous estimation of ofloxacin and ornidazole in Tablet Dosage Form. Indian Drugs.

[13] Patel PU, Suhagia BN, Patel CN, Patel MM, Patel GC, Patel GM (2005). Simultaneous spectrophotometric estimation of gatifloxacin and ornidazole in mixture. Indian J Pharm Sci.

[14] Paramane S, Kothapalli L, Thomas A, Deshpande AD (2006). Simultaneous spectrophotometric estimation of gatifloxacin and ornidazole in tablet dosage form. Indian J Pharm Sci.

[15] Wate SP, Nimje H, Ramtake M (2007). Simultaneous spectrophotometric estimation of gatifloxacin and ornidazole in tablets. Indian J Pharm Edu Res.

[16] Kamble NS, Venkatachalam A (2005). High performance liquid chromatographic determination of ornidazole and ofloxacin in solid dosage forms. Indian Drugs.

[17] Nagavallai D, Sankar AS, Karunambigni AK, Raja MS (2007). Reverse phase-HPLC method for simultaneous estimation of gatifloxacin and ornidazole in tablets. Indian J Pharm Sci.

